# Psychosocial Correlates of Physical Activity Engagement in University Students: A Systematic Review of Resilience and Emotional Processes

**DOI:** 10.3390/healthcare14131929

**Published:** 2026-07-01

**Authors:** Nuria Pérez-Romero, Montserrat Caballero-Cerbán, Silvia San Román Mata

**Affiliations:** 1 Exercise and Rehabilitation Sciences Institute, Postgraduate, Faculty of Rehabilitation Sciences, Universidad Andres Bello, Santiago 7591538, Chile; nuria.perez@unab.cl; 2 Department of Sport Science, Faculty of Management and Health, EADE-University of Wales Trinity Saint David, 29018 Malaga, Spain; 3 Research Unit of Excellence of the University Campus of Melilla (UECUMEL), University Campus of Melilla, University of Granada, 52005 Melilla, Spain; silviasanroman@ugr.es; 4 Department of Nursing, Faculty of Health Sciences, University of Granada, 18071 Granada, Spain

**Keywords:** emotions, college, exercise, sport, adherence

## Abstract

**Highlights:**

**What are the main findings?**

- Positive emotional experiences during physical activity, particularly enjoyment and resilience, were associated with greater exercise adherence, engagement, and participation across university populations.
- Negative emotional states such as boredom and negative emotions were associated with lower adherence, reduced engagement, and lower participation in physical activity.

**What are the implications of the main findings?**

- Exercise programs in educational and recreational settings should incorporate strategies that enhance enjoyment, positive affect, and psychological resilience to improve long-term adherence to physical activity.
- Longitudinal and experimental studies are needed to establish whether these associations represent causal mechanisms underlying long-term exercise adherence.

**Abstract:**

**Background:** Physical exercise is key to good health, but during the transition to university, levels of physical activity often decline. In this context, resilience and emotional intelligence are important psychological resources that may support sustained participation in sport, while exercise-related emotional processes, including affective experiences such as enjoyment, boredom, and anger, may also influence engagement in physical activity. **Objective:** to analyze the relationship between adherence to sports-related physical activity and psychosocial variables such as resilience and emotional processes among university students. **Methods:** This systematic review followed the PRISMA guidelines and was registered in PROSPERO (CRD42025641102). The search was conducted in January 2025 in PubMed, Web of Science and Scopus. Quantitative and qualitative studies evaluating sporting engagement related to resilience or emotional components among university students were selected. Risk of bias was assessed using the Mixed Methods Appraisal Tool. **Results:** five studies with sample sizes ranging from 72 to over 48,000 participants were included. High levels of resilience were associated with more frequent participation in recreational activities and acted as a protective factor against academic stress. Enjoyment is the strongest predictor of behavioral and emotional commitment to sport. Conversely, boredom and negative emotions predict lower levels of future participation. Students with higher emotional responses coped better with psychological barriers and reported greater satisfaction with their performance, which ensures the habit is maintained. **Conclusion:** Resilience and emotional processes appear to be associated with physical activity engagement among university students. However, given the limited number of studies and their heterogeneity, these findings should be interpreted as preliminary and hypothesis-generating. The results suggest the potential relevance of fostering these psychological capacities as part of broader strategies aimed at reducing sedentary behavior and promoting mental wellbeing in university populations.

## 1. Introduction

Physical exercise is defined as a form of planned, structured and repetitive physical activity aimed at improving or maintaining physical fitness [1]. Its importance lies in benefits such as improved cardiorespiratory fitness, reduced depressive symptoms and enhanced self-esteem [2,3,4]. Furthermore, a sedentary lifestyle is associated with poorer indicators of physical and mental health [5] and is linked to sleep disorders and cardiovascular or metabolic risks [2,6]. Furthermore, ceasing regular exercise removes an essential protective factor, exacerbating vulnerability to anxiety and depression at a stage of life when these disorders tend to emerge most frequently [7,8]. Globally, it is estimated that by 2030, inactivity will cause 499 million new cases of non-communicable diseases and mental disorders, at a cost of 47.6 trillion dollars [9]. Therefore, regular exercise is a fundamental strategy for multiple purposes [10,11], as exemplified by the World Health Organization’s initiative to reduce inactivity by 15% through its Global Action Plan [9].

Currently, young people exhibit a high prevalence of sedentary behavior and physical inactivity, largely associated with modern lifestyles [12]. The transition to university life represents a critical period during which the consistency of physical activity habits decreases significantly compared to childhood and adolescence [13]. This reduction is closely linked to high academic demands and rigid timetables, factors that not only limit available time but also increase students’ perceived stress and mental exhaustion [8]. Furthermore, the disproportionate increase in sedentary behavior, driven by the intensive use of screens for study and leisure, has become a major obstacle that directly competes with time allocated to exercise [7,8]. Added to these factors are key psychosocial and contextual barriers, such as low self-efficacy, a lack of intrinsic motivation, and residential or campus environments that are unfavorable to sporting activity [14,15]. Psychosocial factors, meanwhile, appear to be an important aspect of adherence to physical activity, including intrinsic motivation, social support, self-efficacy, resilience and emotional intelligence, amongst others [16,17].

In this regard, resilience, defined as the psychological capacity to recover or “bounce back” quickly from stressful events and adversity [18], acts as a critical mediator that explains approximately 50% of the protective effect of exercise on negative emotions, strengthening stress tolerance and facilitating persistence in healthy habits even under high-pressure conditions [7,8]. In the university population, high levels of resilience act as a buffer that allows for continued participation in sports despite high academic demands, reducing vulnerability to anxiety and promoting positive psychological functioning. Among the university population, high levels of resilience act as a buffer that allows students to continue participating in sports despite demanding academic pressures, reducing vulnerability to anxiety and promoting positive psychological functioning [7,8]. On the other hand, Emotional Intelligence (EI) is defined as the inherent ability to perceive, understand, self-regulate, and use one’s own and others’ emotions as a compass for thought and action [19]. In the sports context, EI involves a meta-emotional capacity that allows students to identify and manage the unique affective responses that arise during exercise, facilitating decision-making and maintaining enthusiasm even when results are not as expected [19,20]. This factor is critical for adherence, as individuals with higher EI, specifically in its trait dimension, better manage psychological barriers and report greater satisfaction with their performance, which ensures long-term maintenance of the habit [20,21].

In addition to the concept of EI as a global construct, EI has been conceptually linked in the literature to emotional experiences such as enjoyment, anger, boredom, and affection, as these serve as the “raw material” upon which emotional capacities operate [22,23]. According to the theory of Levels of Emotional Consciousness, general affect and the impulse to act (such as boredom or enjoyment) form the second level of consciousness, while specific emotions like anger are in the third [23]. A key aspect associated with emotional intelligence is the ability to transform these basic physical sensations into clear and specific emotional concepts through a process called “representational re-description” [23]. Assessing these states can provide information about emotional perception and understanding, which are pillars of EI [22]. This allows us to observe whether the person can identify dimensions such as the level of pleasure or arousal they feel, and whether they understand the reasons behind that emotion [22]. Therefore, the clarity and detail with which someone describes what they feel can provide indirect information about emotional awareness and emotional processing, which are related to emotional intelligence, although they do not constitute a direct and comprehensive measure of it [23].

Emotional intelligence also serves as a critical psychological resource that mitigates academic burnout among college students [24,25], reducing the impact of stress and the physical and mental exhaustion resulting from educational demands [26]. Furthermore, EI has been identified as a fundamental driver for the development of resilience, enabling students to regain their emotional balance and adapt proactively in the face of adversity [27]. This resilience acts as a buffer against the core dimensions of burnout, such as emotional exhaustion and cynicism, improving perceived efficacy and overall well-being [27]. Burnout, in turn, is associated with psychological distress, anxiety, and a severe decline in quality of life [24] and inversely with engagement in health-promoting behaviors; specifically, students with high levels of burnout show lower participation in physical and sports activities, as accumulated stress undermines the intrinsic motivation and energy needed to maintain these healthy habits [28].

Previous studies have examined the relationship between physical activity and mental health, identifying variables such as self-esteem, self-efficacy, and resilience as key mediators of well-being among adolescents and young adults [7,8]. The evidence highlights that high levels of sports participation are associated with reduced perceived stress and anxiety [8,13]. However, a significant knowledge gap persists because most studies are cross-sectional, which prevents the establishment of definitive causal relationships regarding adherence [7]. Furthermore, although resilience has begun to be studied as a protective factor, there is a scarcity of reviews that specifically analyze emotional intelligence in relation to persistence in university sports programs, a stage where the stability of habits tends to decrease and the psychosocial mechanisms that ensure long-term adherence have not been fully elucidated [13,14,15].

Therefore, the overall objective of this review was to analyze the relationship between adherence to sports-related physical activity and psychosocial variables such as resilience and emotional processes among university students. This analysis could aid in decision-making regarding the development of programs that promote sports-related physical activity among university students.

## 2. Materials and Methods

This systematic review followed the PRISMA guidelines (Preferred Reporting Items for Systematic Reviews and Meta-Analyses, [29]). The protocol was previously registered on the PROSPERO platform under the following number: CRD42025641102.

### 2.1. Eligibility Criteria

The PICOT framework (population, intervention, comparison, outcomes, and study design) was used to define the inclusion and exclusion criteria (Table 1).

### 2.2. Sources of Information

The search was conducted using the following databases: PubMed, Web of Science (all databases), and Scopus. Studies that met the eligibility criteria and were identified through other sources (e.g., references from review studies found in databases or through the use of artificial intelligence) were also included following the systematic search to ensure that no relevant studies were overlooked.

### 2.3. Search Strategy

Two authors (N. P.-R. and M. C.-C.) conducted the systematic search, with no restrictions on participant sex, language, or publication date, as of 20 January 2025. To do so, a search strategy based on the PICOT framework was applied, using Medical Subject Headings (MeSH) terms and free-text synonyms (Table 2).

An additional verification search was conducted to assess the comprehensiveness and sensitivity of the original search strategy. This procedure was performed as a supplementary check of robustness and did not alter the predefined review protocol, eligibility criteria, or search strategy in the primary database. The verification search utilized expanded exploratory approaches, including advanced Web of Science functions and semantic expansion techniques assisted by Consensus.ai, to identify studies addressing the research question and under conceptually related terms, such as affective response, self-regulation, and exercise adherence associated with physical activity and exercise participation. The objective was to determine whether potentially eligible studies had been overlooked due to terminological differences. Records identified through this verification process were assessed according to the same eligibility criteria applied in the main review. The guiding question for this search was: among university students participating in sports-related physical activity, are resilience, emotional intelligence, affective response, self-regulation, enjoyment, or specific emotional factors associated with exercise adherence, attendance, retention, compliance, engagement, or continued participation over time?

### 2.4. Selection Process

The studies identified through the various databases were uploaded to the Rayyan.ia web (https://www.rayyan.ai/, accessed on 20 January 2025). This platform allows users to conduct the article selection process for systematic reviews. First, the articles with the highest similarity were identified, and subsequently, one author (N. P.-R.) reviewed each of the similarities, eliminating articles that were duplicates. Following this, the titles and abstracts were read, and two authors (N. P.-R. and M. C.-C.) independently and in parallel assessed their eligibility according to the established criteria using the same software mentioned above. The reference lists of the included articles and of the reviews identified during the search process were also evaluated to select potentially eligible studies. In the event of discrepancies regarding the authors’ decisions, these were resolved by consensus with a third author (S. R.-M.). The selection of the studies comprising the review was recorded using a flowchart, as established by the PRISMA guidelines [29], which detailed the selection process and the corresponding reasons for exclusion.

### 2.5. Data Extraction and Management

For each of the included studies, the following data were identified: study citation (author(s) and year), sample size, participant characteristics (gender, age, fitness level, comorbidities, treatments, and social information [e.g., level of social support, family structure, educational level, socioeconomic status]), description of the activity performed, adherence to physical exercise, assessment of adherence, and factors related to adherence.

Mean values and standard deviation, the percentage for the adherence rate or the analysis done were recorded, considering the period before and after the intervention or exposure, depending on the study type. Data extraction was performed by one author (N. P.-R.) and verified by a second author (M. C.-C.). Each author independently entered the data into a Microsoft Excel spreadsheet (Microsoft Corporation, Redmond, WA, USA). Any conflicts were resolved by consensus with a third author (S. R.-M.).

When the data were not clearly or completely presented in the document, the authors of these studies were contacted up to two times within a two-week period. In the absence of a response or necessary data, the study was excluded from the analysis. Any discrepancies were resolved by consensus with a third author (S. R.-M.). The synthesis was conducted descriptively, separating the data obtained for resilience from that obtained for emotional intelligence.

### 2.6. Risk of Bias and Certainty of Evidence

The risk of bias was assessed using the Mixed Methods Appraisal Tool (MMAT [30]), which allows for the simultaneous evaluation of qualitative, quantitative (randomized, non-randomized, descriptive), and mixed-methods studies. For each included study, the corresponding MMAT criteria category was selected after answering the two mandatory screening questions. The risk of bias was assessed independently by two authors (N. P.-R. and M. C.-C.), with discrepancies resolved through the intervention of a third author (S. R.-M.). This assessment is presented by detailing compliance with each of the five specific criteria of the assigned category, avoiding the use of an overall numerical score, as recommended by the instrument’s manual. To this end, the assessment (yes, no, cannot say) was recorded on a table. The risk of publication bias and certainty of evidence were not assessed since there were fewer than 10 studies per outcome.

## 3. Results

### 3.1. Selection

**Figure 1:**
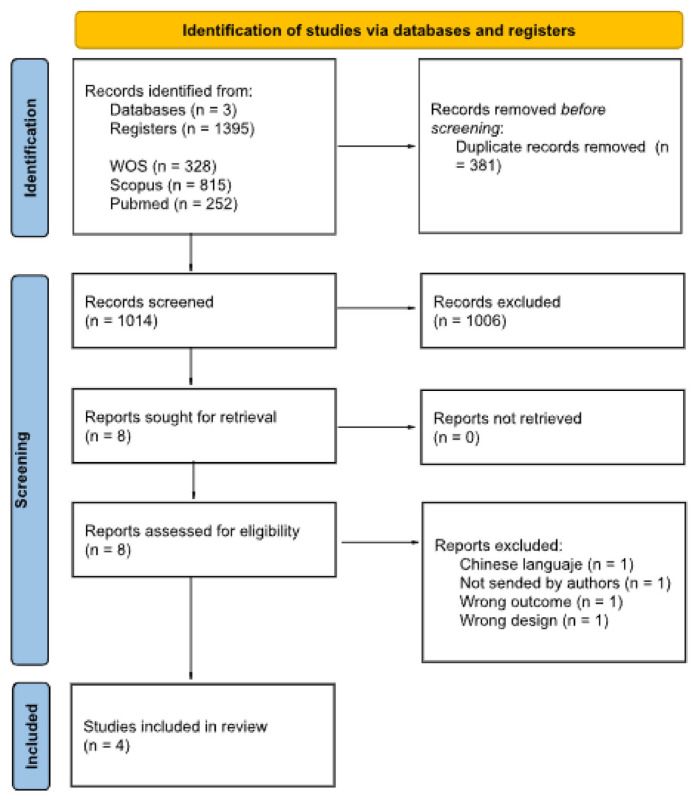
Flow diagram by PRISMA.

Figure 1 shows the PRISMA flow diagram. Initially, 1395 records were identified from the three databases. After removing 381 duplicate records, 1014 records were reviewed during the screening phase, of which 1006 were excluded for failing to meet the criteria. Eight reports were requested for retrieval, excluding four studies (one because it was in Chinese, one because it was not provided by the authors, one because it presented an irrelevant result, and one because it was a cross-sectional study) (Appendix A, Table A1), leaving a total of four studies included in the review. No additional studies meeting the inclusion criteria were identified, supporting the adequacy and sensitivity of the original search strategy.

### 3.2. Characteristics of the Selected Studies

Table 3 shows that the included studies were characterized by quantitative quasi-experimental designs, conducted among young university populations in the United States, with highly variable sample sizes ranging from smaller samples (*n* = 72) to large-scale studies with more than 48,000 participants. Mixed-gender samples aged 17 to 25 years predominated. Physical fitness was assessed in a heterogeneous manner and, in some cases, not reported, although several studies included self-reported measures of moderate and vigorous physical activity. Information on comorbidities was scarce, except in one study that considered the presence of disability, while social variables (educational level, ethnicity, socioeconomic status, family structure, or residence) were described in greater detail in studies with larger sample sizes.

### 3.3. Relationship Between Adherence and Factors

The physical activity practices analyzed in the studies were highly varied and ranged from formal university settings to more informal and recreational activities. In the academic setting, the studies included university physical education classes, such as golf and tennis, which took place in structured environments and focused on skill development. Participation in various recreational activities offered on campus was also considered, such as intramural sports, sports clubs, fitness classes or group exercise with an instructor, open access to facilities, and outdoor adventure activities or outings. Additionally, some studies analyzed exercise under controlled conditions, through 30-min cycling sessions at moderate aerobic intensity, with heart rate monitoring. Finally, several studies focused on physical activity during leisure time, distinguishing between moderate and vigorous activity, and considering the intensity, duration, and usual frequency of students’ exercise.

Adherence or participation was assessed using scales of behavioral commitment, monitoring of physical activity levels, frequency of participation, or future exercise intentions. Consistently, emotional and psychological factors, such as enjoyment, boredom, beliefs about control, and resilience, emerged as key variables associated with adherence, explaining a significant proportion of the variance in physical activity participation, while boredom and negative emotions were associated with lower levels of adherence or future participation.

The results of the analyzed studies show an association between psychosocial factors and adherence to physical activity among university students, although with differences depending on the variable considered (Table 4).

First, the results indicate that frequent participation in university recreational activities positively impacts resilience levels [34]. Outdoor adventure activities showed the strongest relationship (β = 0.114), followed by open recreation (β = 0.087) and intramural sports (β = 0.075).

On the other hand, the studies showed that positive emotional components were significantly associated with higher levels of behavioral adherence and sustained participation in physical activity. Enjoyment emerged as a robust predictor of adherence, both directly and indirectly, explaining a significant proportion of the variance in behavioral and emotional commitment to sports participation. In this regard, enjoyment proved to be a positive and significant predictor of both behavioral commitment (β = 0.178) and emotional commitment (β = 0.244) in golf and tennis classes [31]. Conversely, boredom negatively predicts behavioral (β = −0.250) and emotional (β = −0.214) commitment, serving as a negative predictor of future vigorous physical activity (β = −0.041 to −0.055) [32]. However, there are conflicting results, as the experimental study by Kyral et al. [33] found no significant relationships between positive or negative affect experienced during exercise and the intention to engage in it again in the future.

### 3.4. Risk of Bias and Certainty Assessment

Table 5 presents the assessment of risk of bias. All studies met the established criteria, except for the study by Kyral et al. [33], which did not meet the criterion of sample representativeness with respect to the target population because it used a snowball sampling method.

## 4. Discussion

The objective of this systematic review was to analyze the relationship between adherence to sports and physical activity and two key psychosocial variables (resilience and emotional processes) in the university population. Overall, the results indicate considerable heterogeneity and limited evidence, although the studies included a large number of participants. Within this limited evidence, the findings suggest that these factors may be related to participation in and continuation of sports and physical activity during college years. However, the findings should be interpreted with caution, as resilience was assessed more directly in the included studies, while emotional intelligence is primarily represented through emotional variables related to the exercise experience, such as enjoyment, boredom, positive and negative effect, and emotional commitment, which should be understood as indicators of emotional and affective processes related to exercise experience rather than as direct measures of emotional intelligence.

Regarding resilience, the results show a positive association between this variable and physical activity (in just one study, but with 48,000 participants). These findings suggest that resilience may be associated with a greater capacity to cope with academic demands, stress, and negative emotions [10,35]. These findings are consistent with previous literature that has highlighted the relationship between physical activity, mental health, and mediating psychosocial variables. White et al. [7] emphasize that the association between physical activity and mental health can be explained, at least partially, by psychological and social mediating and moderating mechanisms. Along these same lines, Martín-Rodríguez and González-Prieto [8] note that physical activity among college students is associated with lower levels of perceived stress and better indicators of mental health. Furthermore, frequent participation in college recreational activities was associated with higher levels of resilience, particularly in outdoor adventure and recreational activities. These findings suggest that physical and sports activities, particularly when conducted in participatory and recreational settings, may be associated with greater psychological resilience, potentially through mechanisms such as social support, the development of coping skills, and improved psychological well-being associated with sports participation [36].

However, it cannot be definitively stated that physical activity leads to increased resilience or that resilience alone guarantees greater adherence. The relationship may be bidirectional: on the one hand, more resilient students may have greater resources to maintain their sports participation in the face of stress, lack of time, or academic pressure [37,38]; on the other hand, regular participation in physical activities could promote the development of resilience by providing a social environment that reinforces feelings of competence and social acceptance [39]. This interpretation is particularly prudent given that some of the available evidence comes from a design that does not allow for the establishment of causality. From a theoretical perspective, resilience may be important not because it increases participation per se, but because it could help sustain participation when barriers emerge.

About emotional intelligence, the results should be interpreted with greater caution, as the included studies did not assess this construct directly using specific emotional intelligence instruments. However, several studies analyzed emotional components linked to physical and sports activities, such as enjoyment, boredom, positive affect, negative affect, and emotional commitment. These indicators provide insight into the role of emotional processes in adherence to physical activity, although they should not be completely conflated with emotional intelligence as an overarching construct. In this sense, the emotional aspects identified reflect affective experiences and responses during sports participation, rather than emotional intelligence as a dispositional construct. Enjoyment emerged as the most consistently reported predictor of behavioral and emotional commitment across the included studies, highlighting its role as an affective experience associated with intrinsic motivation and sustained participation [40]. Conversely, boredom emerges as an affective experience associated with lower adherence, reinforcing the idea that emotional regulation is crucial for overcoming the intrinsic obstacles of a sports routine [41]. Therefore, the findings suggest that students who perceive physical and sports activities as enjoyable, satisfying, and meaningful tend to report higher levels of engagement and continued participation.

These findings can be linked to self-determination theory, as continued physical activity has been associated with autonomous motivation, personal satisfaction, and positive experiences during the activity [41]. In this regard, Ntoumanis [42] highlights the importance of autonomous motivation in promoting sustained physical activity. Thus, the findings of this review suggest that enjoyment should not be viewed as a secondary aspect, but rather as a potentially important emotional correlate of commitment and retention in university sports. Conversely, boredom emerges as an emotional factor consistently associated with lower adherence, indicating that sports experiences perceived as monotonous, unappealing, or disconnected from students’ interests may be associated with lower engagement and a greater likelihood of dropout [43]. From this perspective, the emotional dimension of participation is fundamental; it is not enough to simply offer physical activity, but rather it is necessary to create motivating and emotionally positive experiences.

About positive and negative affect, the results were less conclusive. Kyral et al. [33] found no significant relationship between the affect experienced during a single session of aerobic exercise and future intention to exercise. This finding suggests that a one-time affective experience may not be sufficient to predict future adherence, especially when adherence is measured by intention rather than by sustained behavior over time. Furthermore, future intentions to exercise may depend on other factors, such as prior habits, self-efficacy, perceived competence, motivation, time availability, or the social context of exercise [44]. Therefore, no clear conclusions can be drawn regarding the relationship between emotional processes and exercise adherence based on the studies included in this review. The limited number of eligible longitudinal studies and the heterogeneity in how emotional constructs were assessed prevent robust inferences about the role of emotional intelligence in sustaining physical activity over time. Nevertheless, evidence from related literature suggests that emotional intelligence may be a relevant psychological construct associated with physical activity engagement and maintenance, although it was not directly assessed in the included studies. For example, Ubago-Jiménez et al. [19], note that physical activity and sports may be related to the development of emotional intelligence. Similarly, An et al. [20] highlights the relationship between emotional intelligence in sports, self-efficacy, and exercise adherence among college students. In this regard, emotional intelligence may help students better identify, understand, and regulate the emotions associated with physical activity, thereby facilitating greater consistency.

### 4.1. Limitations

This review has several limitations that should be considered when interpreting the findings. First, only a small number of studies met the inclusion criteria (*n* = 4), limiting both the generalizability and certainty of the available evidence. Furthermore, the included studies exhibited substantial methodological heterogeneity in terms of study design, sample size, physical activity modalities, and the instruments used to assess adherence and psychosocial variables. This variability limited comparability across studies and precluded a quantitative synthesis of the data. Importantly, the limited number of studies and their heterogeneity appear to reflect the current state of the literature rather than shortcomings in the review process, highlighting the scarcity of research specifically examining physical activity adherence in relation to resilience and emotional intelligence among university students. Consequently, the findings should be interpreted as a narrative synthesis intended to map existing evidence and identify research gaps.

A further limitation concerns the assessment of emotional constructs. Several studies assessed specific emotional or affective experiences during physical activity, such as enjoyment, boredom, or negative affect, rather than emotional intelligence as a unified construct. Therefore, conclusions regarding emotional intelligence should be interpreted with caution and considered exploratory. In addition, some studies relied on self-reported measures of physical activity, which may be subject to recall and social desirability bias. The predominance of studies conducted in the United States further limits the cultural and contextual generalizability of the findings.

Another important limitation concerns the availability of eligible evidence. Although a larger body of literature exists examining relationships between physical activity and psychological variables such as resilience and emotional experiences, much of this research is based on cross-sectional designs. These studies frequently explore associations or mediation models but do not assess adherence over time and, therefore, did not meet the eligibility criteria of the present review. As a result, only a small number of studies were eligible for inclusion.

This scarcity of eligible studies reflects the methodological and logistical challenges involved in conducting longitudinal research with repeated follow-up assessments, which are necessary to evaluate actual adherence to physical activity. Consequently, the current evidence base remains limited, and the findings of this review should be interpreted considering the relatively small number of longitudinal investigations available. Finally, the small number of included studies also limited the possibility of formally assessing publication bias, which should be considered when evaluating the robustness of the evidence base.

### 4.2. Future Research

Overall, the results of this review suggest that adherence to physical activity among college students should be understood as a multifactorial process. Resilience may promote persistence in the face of stress, academic demands, and negative emotions; meanwhile, emotional components linked to emotional intelligence, particularly enjoyment and the regulation of negative experiences such as boredom, may influence commitment and continuity of participation. Future research should examine more specifically the relationship between resilience, emotional intelligence, and adherence to physical activity among college students. To this end, it would be necessary to conduct longitudinal studies and experimental interventions to determine whether strengthening resilience and emotional intelligence improves retention in college sports programs.

Since emotional intelligence was not directly assessed in all studies, it is necessary to exercise caution and consider these findings as an initial approximation of the role of emotional processes in college sports adherence. Nevertheless, the findings highlight a potentially important line of research. While previous studies have extensively explored associations between emotional variables and physical activity using cross-sectional designs, future longitudinal and experimental studies are needed to determine whether these factors play a causal role in exercise adherence over time. Such research would help clarify the extent to which the relationships suggested by the existing literature reflect true mechanisms underlying adherence and would contribute to a more robust understanding of this emerging field.

Likewise, it would be advisable to use validated instruments that directly measure emotional intelligence, distinguishing it from other emotional indicators such as enjoyment, boredom, or affect. It would also be beneficial to combine subjective and objective measures of adherence, including questionnaires, attendance records, weekly frequency of participation, duration of participation, and actual dropout rates. Finally, studies are needed in different cultural and university contexts to verify whether these results hold true across diverse populations.

### 4.3. Practical Implications

From an applied perspective, the results suggest that universities should design physical activity programs that go beyond simply providing access to sports facilities and also incorporate psychological and emotional components. Specifically, it may be beneficial to promote activities that build resilience, foster a sense of achievement, enhance enjoyment, and reduce boredom during participation. Universities may also consider strategies that help students cope with academic stress, manage exercise-related frustration, and maintain positive emotional experiences during physical activity. This approach provides a more comprehensive perspective on physical activity adherence, considering not only sports behavior but also the psychological and emotional resources that may support sustained participation over time. However, these implications should be interpreted cautiously, given the limited number of studies available, underscoring the need for further research to strengthen the evidence base in this area.

## 5. Conclusions

In summary, this systematic review indicates that resilience is associated with physical activity participation among university students. Emotional and affective variables, such as enjoyment and boredom, also appear to be related to engagement in physical activity. However, these findings are based primarily on observational evidence, and causal interpretations cannot be established. Evidence regarding emotional intelligence should be interpreted with caution, as it was not consistently or directly measured across the included studies, and is often represented through related affective indicators rather than a unified construct. Overall, the findings should be considered preliminary and hypothesis-generating. Further longitudinal and intervention studies are needed to clarify the role of resilience and emotional processes in physical activity engagement in university populations.

## Figures and Tables

**Table 1:** Eligibility criteria.

	Inclusion Criteria	Exclusion Criteria
P	University students	Any related medical condition
I	Sports-related physical activity	No exclusion criteria are established
C	Comparison of adherence versus non-adherence to exercise, assessed through adherence rates, such as attendance rates by session or participant	No type of adherence assessment will be excluded, and an attempt will be made to establish a common measure where possible
O	Resilience and emotional intelligence associated with adherence as described in the studies themselves. Studies that did not measure emotional intelligence as a general measure, but rather a specific aspect of it (e.g., a particular emotion), were included.	No variables will be excluded
T	Experimental or non-experimental longitudinal studies, and qualitative studies	Studies that do not include results, such as conference posters or abstracts without full-text documents

**Table 2:** Search strategy for each database.

Database	Data Search Strategy
Pubmed	(((((resilience[Title/Abstract]) OR (“Emotional Intelligence”[Title/Abstract])) OR (emotion*[Title/Abstract])) AND (((((adherence) OR (compliance)) OR (retention)) OR (engagement)) OR (“Drop out”))) AND (((sport[Title/Abstract]) OR (“physical exercise”[Title/Abstract])) OR (“physical activity”[Title/Abstract]))) AND (((college[Title/Abstract]) OR (university[Title/Abstract])) OR (student*[Title/Abstract]))
Web of Science	1: TS = (resilience) 2: TS = (“Emotional Intelligence”) 3: TS = (emotion*) 4: ALL = (adherence) 5: ALL = (Compliance) 6: ALL = (Retention) 7: ALL = (Engagement) 8: ALL = (“Drop out”) 9: TS = (sport) 10: TS = (“physical exercise”) 11: TS = (“physical activity”) 12: TS = (college) 13: TS = (university) 14: #1 OR #2 OR #3 15: #4 OR #5 OR #6 OR #7 OR #8 16: #9 OR #10 OR #11 17: TS = (student*) 18: #17 OR #12 OR #13 19: #18 AND #16 AND #15 AND #14
Scopus	( TITLE-ABS ( resilience ) OR TITLE-ABS ( “Emotional Intelligence” ) OR TITLE-ABS ( emotion* ) ) AND ( ALL ( adherence ) OR ALL ( compliance ) OR ALL ( retention ) OR ALL ( engagement ) OR ALL ( “Drop out” ) ) AND ( TITLE-ABS ( sport ) OR TITLE-ABS ( “physical exercise” ) OR TITLE-ABS ( “physical activity” ) ) AND ( TITLE-ABS ( college ) OR TITLE-ABS ( university ) OR TITLE-ABS ( student* ) )

Note. The term “emotion*” was intentionally used as a broad sensitivity-enhancing search term because emotional experiences, affective responses, emotion regulation, and emotion use are considered core dimensions or manifestations of Emotional Intelligence across major theoretical models; student* = is used as a truncation symbol to retrieve both singular and plural forms, such as “student” and “students”.

**Table 3:** Study characteristics.

Reference	Study Design	Country	Sample	Gender	Age M (SD)	Physical Fitness Level	Comorbidities	Social Information
Garn et al. 2017 [31]	Short-term longitudinal quasi-experimental study over 15 weeks (pretest–posttest)	USA (Louisiana State University)	202 university students enrolled in elective golf (5 classes) and tennis (4 classes) courses, with class sizes ranging from 18 to 24 students	109 men and 92 women	20.73 (1.49)	NR	NR	Ethnicity: 76% Caucasian/European, 10% Black/African American, and other ethnic minorities (Asian and Hispanic)
Garn et al. 2022 [32]	Longitudinal study with a full mediation model (three waves of data collection)	USA	586 undergraduate students (402 participants [69%] completed all three waves, 95 [16%] completed two waves, and 89 [15%] completed one wave only)	65% women, 35% men	19.44 (1.43)	Global Physical Activity Questionnaire (GPAQ). Seventy percent of the sample reported engaging in at least 150 min of moderate-to-vigorous LTPA per week; 210 min of moderate LTPA and 98 min of vigorous LTPA weekly across the three time points	NR	Educational level: Seniors 55%, Juniors 38%, Sophomores 6%; Ethnicity: White/Caucasian 68%, Black/African American 18%, Other ethnicities 14%
Kyral et al. 2019 [33]	Non-randomized experimental study (single session)	USA	72 university students	41 men, 31 women	17 to 25 years	Exercise Participation Survey classified participants as active (*n* = 38) or inactive (*n* = 34)	NR	Ethnicity: Caucasian (*n* = 25), Black (*n* = 2), Asian (*n* = 2)
Soria et al. 2022 [34]	Quasi-experimental study using propensity score matching	USA	48,232 undergraduate university students	63.6% women, 35.3% men, and 1% gender non-conforming	Under 24 years (91.8%) and over 24 years (8.2%)	NR	Fourteen percent (6761 students) reported having a disability; 86% (41,471 students) reported no disabilities	Socioeconomic level: 16.6% reported family incomes of USD 100,000–149,999, 12.5% over USD 200,000 annually, and 15.8% did not know parental income; Family structure and residence: 42.3% lived in university housing, 10% with parents/guardians, and 6.4% off campus with partner/spouse/children; 32.6% were first-generation students; Academic level: 23.2% first-year, 22.0% second-year, 25.5% third-year, and 29.3% fourth-year or above; Fields of study: Business (15.2%), Social Sciences (14.3%), Engineering (10.8%), and Health (10.1%)

**Table 4:** Adherence results.

Reference	Description of the Activity Performed or Evaluated	Adherence Assessment	Assessment of Factors Related to Adherence	Exercise Adherence Results Related to Factors
Garn et al. 2017 [31]	Structured golf and tennis classes	Skinner Engagement Scales: Assessed changes in effort and attention (behavioral engagement) throughout the semester	Achievement Emotions Questionnaire (AEQ): Measured enjoyment, anger, and boredom during class	Enjoyment positively predicted changes in behavioral engagement (β = 0.178, *p* < 0.01) and emotional engagement (β = 0.244, *p* < 0.01); boredom negatively predicted changes in behavioral engagement (β = −0.250, *p* < 0.01) and emotional engagement (β = −0.214, *p* < 0.01); anger was not significantly related to changes in engagement; the model explained 39% of the variance in behavioral engagement changes and 40% in emotional engagement; extrinsic value beliefs indirectly predicted behavioral adherence through enjoyment (β = 0.062, *p* < 0.05)
Garn et al. 2022 [32]	Moderate- and vigorous-intensity leisure-time physical activity (LTPA)	Monitoring of physical activity and sedentary behavior levels throughout the semester. Global Physical Activity Questionnaire (GPAQ): Weekly minutes of moderate and vigorous activity reported	8-item scale: Measured enjoyment and boredom toward leisure-time physical activity	Control beliefs were robust predictors of moderate LTPA (β = 0.240 to 0.255, *p* < 0.01) and vigorous LTPA (β = 0.160 to 0.217, *p* < 0.01); boredom negatively predicted future vigorous LTPA (β = −0.041 to −0.055, *p* < 0.05); initial value through enjoyment predicted moderate LTPA at wave three (IDE = 0.021, 95% BCI [0.005, 0.042]); the model explained up to 42.6% of the variance in vigorous LTPA at the end of the study
Kyral et al. 2019 [33]	A 30-min aerobic exercise session on a cycle ergometer in which heart rate was monitored to maintain moderate intensity (65–75%) of age-predicted maximum heart rate	Exercise Intention Index: Measured students’ intention to exercise in the future following the session	Positive and Negative Affect Scale (PANAS): Assessed well-being/affect during exercise	No significant differences between active and inactive students in positive affect (U = 664.5, *p* = 0.834) or negative affect (U = 720, *p* = 0.397); men scored significantly higher than women (U = 440, *p* = 0.026); no significant relationship between positive affect and future exercise intention (rs = 0.098, *p* = 0.415) or between negative affect and intention (rs = −0.058, *p* = 0.629)
Soria et al. 2022 [34]	The study evaluated participation in five specific types of recreational activities offered on campus: (1) instructor-led group fitness or exercise classes; (2) intramural sports; (3) open recreation; (4) outdoor adventure activities or trips; and (5) sport clubs. The assessment focused on participation frequency, defining “active participants” as those engaging in these activities “often” or “most of the time.”	MSL Survey: Frequency of active participation (“often” or “always”) in campus programs	CD-RISC-10: Brief version of the Connor-Davidson Resilience Scale	Impact on resilience (β): Outdoor adventure activities β = 0.114, *p* < 0.001; open recreation β = 0.087, *p* < 0.001; intramural sports β = 0.075, *p* < 0.001

Note: LTPA = leisure-time physical activity.

**Table 5:** Risk of bias by MMAT.

	Screening Questions (for All Types)		3. Quantitative Nonrandomized				
References	S1. Are There Clear Research Questions?	S2. Do the Collected Data Allow Addressing the Research Questions?	3.1. Are the Participants Representative of the Target Population?	3.2. Are Measurements Appropriate Regarding Both the Outcome and Intervention (or Exposure)?	3.3. Are There Complete Outcome Data?	3.4. Are the Confounders Accounted for in the Design and Analysis?	3.5. During the Study Period, Is the Intervention Administered (or Exposure Occurred) as Intended?
Garn et al., 2017 [31]	YES	YES	YES	YES	YES	YES	YES
Garn et al., 2022 [32]	YES	YES	YES	YES	YES	YES	YES
Kyral et al., 2019 [33]	YES	YES	NO	YES	YES	YES	YES
Soria et al., 2022 [34]	YES	YES	YES	YES	YES	YES	YES

## Data Availability

No new data were created or analyzed in this study. Data sharing is not applicable to this article.

## Appendix A

**Table A1:** Study selection.

Reference	Inclusion	Reason
Garn et al., 2017 [31]	YES	
Garn et al., 2022 [32]	YES	
Kowalska et al., 2021 [45]	NO	The aim of the study was to assess emotional status and the level of sense of coherence in the context of physical activity among physiotherapy students, and to investigate how the emotional status of participating students changed after two years of study and which factors were associated with mood disorders in the group of students who were about to complete their studies. Wrong outcome because it evaluates BDI, PSQ, and SOC-29.
Kyral et al., 2019 [33]	YES	
Liu et al., 2024 [46]	NO	Cross-sectional study.
Sick et al., 2023 [47]	NO	Requested from the authors but not received.
Soria et al., 2022 [34]	YES	
Zhang et al., 2024 [48]	NO	The article is in Chinese.
